# The Lay Contributor

**Published:** 1920-06-05

**Authors:** 


*To the Editor of* The Hospital.

### The Lay Contributor.

*To the Editor of* The Hospital.

Sir,—I have wondered that among the many incidents recorded of Sir Henry Burdett's work little or no mention has been made in connection with what always appears to me to have been a feature of the late Editor's policy in regard to institutional life—namely, his willingness to hear and consider the voice of the layman.

Many a valuable paragraph and article have found their way into the columns of The Hospital, thanks to the sympathetic and broad mind of Sir Henry Burdett, who would never "turn down" a lay contributor merely because he was such, but was ever ready to encourage well-informed public criticism, interest, and opinion of hospital administration and undertakings; witness the remarkable series of articles which appeared under the title of "Hospitals from the Patients' Point of View."

Yours faithfully,

Hospital Worker.

